# Midterm Aortic Neck Evolution after EndoSuture Aneurysm Repair: a Single Centre Retrospective Analysis

**DOI:** 10.1016/j.ejvsvf.2025.09.002

**Published:** 2025-09-08

**Authors:** Raffaele Pio Ammollo, Nicolas Mauchien, Alexandre Oliny, Marine Bordet, Nellie Della Schiava, Antoine Millon

**Affiliations:** a Department of Vascular and Endovascular Surgery, Hospices Civils de Lyon, Louis Pradel University Hospital, Bron, France; b Aortic Centre, Hôpital Marie Lannelongue, GHPSJ, Le Plessis-Robinson, France; c Université Claude Bernard Lyon 1, Villeurbanne, France; d Université de Lyon, INSA Lyon, LGEF, EA682, Villeurbanne, France

**Keywords:** EndoAnchors, Endovascular aneurysm repair, Hostile aortic neck, Type 1 endoleaks

## Abstract

**Objectives:**

Long term results from large international registries have shown satisfactory results in terms of type 1 endoleak (EL1a) prevention and sac shrinkage using EndoSuture aneurysm repair (ESAR) in patients with a hostile aortic neck; however, little is known about the midterm behaviour of the aortic neck after ESAR.

**Methods:**

This study retrospectively analysed the aortic neck evolution and overall results of ESAR procedures performed at this institution between September 2017 and August 2020. Patients with a hostile aortic neck, and or who were unfit for elective open repair, and or presented with abdominal aortic target vessel or iliac anatomy unsuitable for a fenestrated endograft (FEVAR), and or for whom there was insufficient time for FEVAR manufacturing were included.

**Results:**

Twenty-three patients were included (male 22/23, 96%; median age 75 years, range 58–87 years), and were followed up for 36.5 ± 16.3 months. Technical and procedural success rates were 100% and 96%, respectively. No aortic rupture or dissection was encountered peri-operatively and no displacement, migration, or unachieved penetration of the EndoAnchors was observed. The median operating time was 145 (range 87–236) minutes. No aortic neck dilation was observed at six, 12, 24, and 36 months. There was no persistent or new EL1a or limb occlusion. The 30 day and one year mortality rate was 0%. Six non-aneurysm related deaths were observed during follow up (26%). The overall survival at one, two, and three years was 100%, 100%, and 74%, respectively.

**Conclusion:**

This analysis of aortic neck evolution three years after ESAR suggests that EndoAnchors may help prevent aortic neck and suprarenal aortic dilatation in the midterm, without re-interventions for type EL1a. ESAR is a feasible procedure in patients with hostile aortic neck, and/or who are unfit for open surgery, and/or in whom anatomical or technical constraints prevent the use of FEVAR.

## HIGHLIGHTS

• Despite mounting evidence and five years of follow up data coming from international registries, EndoSuture aneurysm repair (ESAR) has failed to gain popularity in vascular practice.
• This study is the first to report 36 months of data on aortic neck evolution after ESAR.
• It found that this is a feasible and safe procedure, valuable in patients with hostile neck, and or unfit for open surgical repair and or with technical constraints for fenestrated endovascular repair.

## Introduction

Endovascular aortic repair (EVAR) is currently the treatment of choice for abdominal aortic aneurysms (AAAs), for cases with suitable anatomy and reasonable life expectancy.^1^ Proximal stent graft sealing depends on aneurysm neck characteristics, such as length, diameter, shape, and angulation, as well as the presence of calcification or thrombus.^2^ Hostile neck anatomy has been shown to be a predictor of type 1a endoleaks (EL1a), re-intervention, and aneurysm related one year death.^3^^,^^4^

Since its introduction into clinical practice, the safety and durability of EVAR in the presence of suboptimal anatomical features have increased because of technical progress regarding endograft quality and corollary armamentarium, including EndoSuture aneurysm repair (ESAR). The Heli-FX EndoAnchor System (Medtronic, Minneapolis, USA) is designed to secure the fabric of an Endurant II/IIs stent graft (Medtronic) to the aortic neck wall.

In a previous propensity score analysis by this group comparing ESAR and fenestrated endovascular aneurysm repair (FEVAR), ESAR achieved comparable midterm outcomes to FEVAR in terms of EL1a, aneurysm shrinkage, and aneurysm related mortality, representing a good off the shelf alternative to FEVAR for technical constraint cases.^5^ However, the recent European Society for Vascular Surgery aortic guidelines highlight the need for more robust data on the long term durability of ESAR, recommending its use only within approved research studies.^1^

Therefore, this study aimed to analyse aortic neck evolution and report the midterm results of a single centre experience with ESAR in patients with a hostile aortic neck, and/or a contraindication to open surgery, and/or for whom anatomical and technical constraints limited the use of FEVAR.

## Materials and methods

### Study design and patient selection

This retrospective analysis included all consecutive patients who underwent ESAR procedures at a single tertiary referral centre between September 2017 and August 2020. The patients were followed up until May 2024. This study was conducted in accordance with the principles of the Helsinki Declaration. All patients were initially included in a previous trial^5^ approved by the ethics committee of the Hospices Civils de Lyon (21_309). The retrospective analysis of their data carried out for this study did not require further approval by an ethics committee.

Patients with degenerative AAAs presenting with a hostile proximal neck, contraindications to elective open repair, abdominal aortic target vessel or iliac anatomy unsuitable for FEVAR, and or insufficient time for FEVAR manufacturing were included.

Hostile necks were defined by the presence of at least one of the following criteria: short neck length ≤10 mm, measured between the lowest renal artery and the point at which the aortic diameter reached >28 mm; angulation ≥60°; width >28 mm; conical (defined as an increase in diameter >10% compared with the immediate infrarenal diameter over the first 10 mm beyond the lowest renal artery).^6^^,^^7^ ESAR was not performed in patients presenting with circumferential calcifications and or thrombus >2 mm in thickness over >50% of the circumference, or in case of asymmetric neck bulges, as those are risk factors for EndoAnchor penetration failure.

Patients treated with ESAR for causes other than degenerative AAAs (infective native, inflammatory, pseudoaneurysms) were excluded from the analysis, as well as those without a one month post-operative computed tomography angiography (CTA) scan, as the latter was used as a baseline examination to minimise the measurement bias of the aortic neck dilation due to stent graft oversizing.

### Surgical procedure

All ESARs were performed by the same experienced surgeon as primary deployments for EL1a prevention in a hybrid room, under general anaesthesia, in a non-emergency setting, using Endurant II/IIs stent grafts (Medtronic) and the EndoAnchor Heli-FX System (Medtronic).^8^ No ESAR was performed as a correction of previous stent graft failure for EL1a. According to the aortic diameter on the pre-operative computed tomography angiogram (CTA), four (aortic diameter ≤29 mm) to six or eight (aortic diameter >29 mm) EndoAnchors were deployed systematically around the aortic circumference. For EL1a cases on completion angiography, additional EndoAnchors were deployed below the first row. Technical success was defined as successful deployment of the stent graft and the desired number of EndoAnchors. Procedural success was defined as successful deployment without EL1a on completion angiography.

### Follow up

CTAs, followed by clinical examinations, were performed at one, six, and 12 months, and annually thereafter until May 2024. Procedural, radiological, and follow up data of the included patients were collected retrospectively from the electronic medical records. Post-operative CTAs were analysed on image reconstruction software with automatic centreline extraction (EndoSize, Therenva SAS, Rennes, France).

### Endpoints

**Figure 1:**
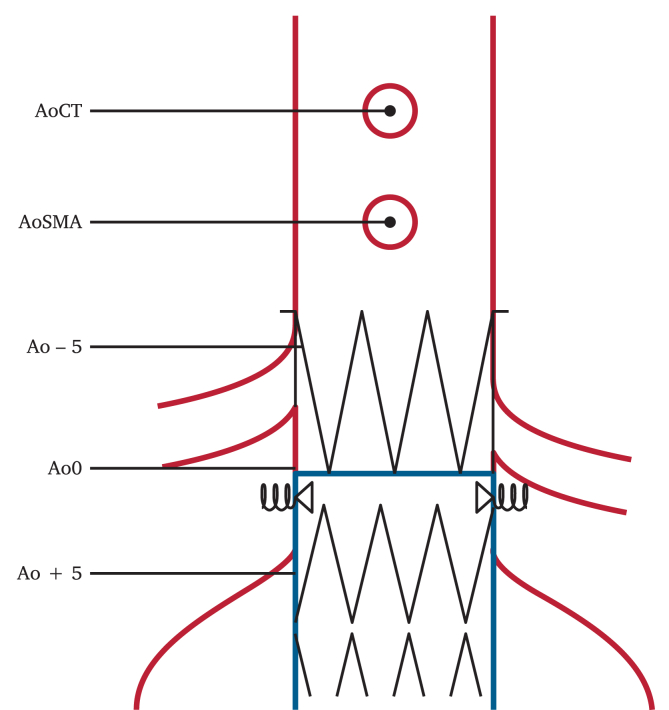
Schematic representation of aortic diameter measurements at different levels. AoCT = central point of the ostium of the coeliac trunk; AoSMA = central point of the ostium of the superior mesenteric artery; Ao-5 = 5 mm above the inferior border of the ostium of the lower renal artery; Ao0 = at the inferior border of the lower renal artery; Ao+5 = 5 mm below the inferior border of the ostium of the lower renal artery. Blue = Endurant II/IIs graft; black = Endurant II/IIs and EndoAnchor stents; red = artery.

The primary endpoint was aortic neck dilation at the inferior border of the lowest renal artery (Ao0) level at six, 12, 24, and 36 month follow ups. To assess aortic neck diameter, inner to inner aortic diameters on anteroposterior and lateral projections were measured at the following levels: central point of the ostium of the coeliac trunk (AoCT), central point of the ostium of the superior mesenteric artery (AoSMA), 5 mm above the inferior border of the ostium of the lowest renal artery (Ao-5), Ao0, and 5 mm below the inferior border of the ostium of the lower renal artery (Ao+5; Fig. 1). The change in diameter was expressed as the difference in diameter between the last available CTA and the one month post-operative CTA, which was used as the baseline examination. A diameter expansion of >2.5 mm reflected aortic neck dilation.^9^

The secondary endpoints were suprarenal (AoCT, AoAMS, and Ao-5) and infrarenal (Ao5) aortic neck dilation, presence of EL1a during follow up, aortic neck related re-interventions, AAA related re-interventions, AAA sac behaviour, and overall and AAA related death. To evaluate AAA sac behaviour, the maximum diameter was compared with the one month post-operative CTA. Sac shrinkage was defined as a decrease of >5 mm, sac stability as a variation in a ±5 mm range, and sac expansion as an increase of >5 mm.

### Statistical analysis

Data collection and analysis were conducted using Microsoft Excel (Microsoft Corporation, Redmond, USA; version 16.96.1). Descriptive statistics were expressed as mean ± SD or median (range) for quantitative variables and as number and percentage for qualitative variables. Due to the small sample size, no exhaustive inferential analysis could be performed.

## Results

### Patients’ characteristics

Twenty-three patients were included in the analysis; 22 (96%) were male and the median age was 74.5 (58–87) years. The main comorbidities and pre-operative anatomic characteristics are reported in Table 1.

**Table 1:** Demographics, patient comorbidities, and anatomic characteristics (*n* = 23).

Variable	Value
*Demographics*	
Male	22 (96)
Age – years	74.5 (58–87)
BMI – kg/m^2^	28.2 (21.2–36.9)
*Comorbidities*	
Arterial hypertension	13 (57)
Active smoking	13 (57)
Dyslipidaemia	11 (48)
CAD	9 (33)
COPD	3 (13)
Diabetes	2 (9)
*Anatomical characteristics*	
Suprarenal angle	16 ± 10.9 (2–45.2)
Infrarenal angle	27.1 ± 14.1 (3–61.4)
Neck length	9.9 ± 3 (4–15)
*Diameters – mm*	
AoCT	25.8 ± 2.3 (22.6–31.4)
AoSMA	26.1 ± 3.2 (22–33.4)
Ao-5	23.8 ± 3.1 (18.5–29.7)
Ao0	23.5 ± 2.4 (17.4–27)
Ao+5	24.8 ± 2.7 (19.3–32.1)
Maximum aortic diameter	58.4 ± 9.3 (50.2–91)
Thrombus and or calcification >50% – *n*	0

BMI = body mass index; CAD = coronary artery disease; COPD = chronic obstructive pulmonary disease; SD = standard deviation; AoCT = central point of the ostium of the coeliac trunk (CT); AoSMA = central point of the ostium of the superior mesenteric artery (SMA); Ao-5 = 5 mm above the inferior border of the ostium of the lowest renal artery; Ao0 = at the inferior border of the lowest renal artery; Ao+5 = 5 mm below the inferior border of the ostium of the lowest renal artery.

Data are shown as *n* (%), median (range), or mean ± SD (range), unless otherwise shown.

Aortic neck hostility criteria leading to ESAR were: short neck in 10 cases; short and conical neck in six cases; conical neck in four cases; and angulated neck in one case. No patient had a large aortic neck. Two frail patients did not present hostility criteria (neck lengths of 12 mm and 15 mm) but were treated by ESAR due to severe clinical comorbidities and lack of manufacturing time for FEVAR. Contraindications for elective open repair were: five heart failure or coronary heart disease, four respiratory insufficiency, three hostile abdomen, five morbid obesity, three chronic kidney disease (GFR of ≤30 mL/min/1.73 m^2^), and three patients aged >80 years. Patients were also considered anatomically unsuitable for FEVAR: 10 owing to target vessel anatomy (stenosis, diameter <4 mm, multiple accessory renal arteries), four owing to a shaggy aorta (defined as the presence of extensive thrombus with irregular surface in the suprarenal aorta) and the associated risk of solid organ infarction during target vessel catheterisation, one owing to coeliac aortic dissection, two owing to iliac access issues (calcified, tortuous, and or <7 mm iliac arteries), as well as six AAAs measuring >70 mm, for which the risk of rupture did not allow for a wait for the customised FEVAR device.

The mean follow up was 36.5 ± 16.3 months, with an overall completion of the follow up of 83%, 91%, 91%, and 62% at 6, 12, 24, and 36 months, respectively.

### Peri-operative results

The median operating time was 145 (87–236) minutes. The median number of EndoAnchors deployed per procedure was seven (range: five – ten); no displacement, migration, or failed penetration of the EndoAnchors was observed. Overall, the technical and procedural success rates were 100% and 96%, respectively. No aortic rupture or dissection was encountered peri-operatively. Supplementary procedures during the primary intervention were necessary in seven patients: proximal cuff plus renal artery stenting to treat intra-operative EL1a in one patient, common femoral prosthetic grafting to gain a safe femoral access in one, iliofemoral junction covered stenting for preclosing failure in two, iliac limb stents on a narrow aortic bifurcation or calcification in two, renal artery stenting because of accidental lowest renal artery coverage in one, and bilateral iliac branch device deployment in one. One patient required an intensive care unit stay of one night. The median length of hospital stay was two (range: two – nine) days.

### Primary endpoint: aortic neck dilation at Ao0 during follow up

No major aortic neck dilation was observed at Ao0 level on the available CTAs at six, 12, 24, and 36 months (Table 2). Mean (range) aortic diameter variations were 0.14 (−2.9–2) mm, 0.65 (−1.8–4.1) mm, 0.08 (–4 - 2.3) mm, −0.58 (–5 - 1.9) mm at six, 12, 24, and 36 months, respectively.

**Table 2:** Aortic neck diameter variations at the level of the lowest renal artery (Ao0) during follow up.

Variable	6 months	12 months	24 months	36 months
Available CTAs in the patient sample	18/23 (78)	21/23 (91)	21/23 (91)	15/23 (65)
Ao0 stability or reduction in the total of available CTAs	18/18 (100)	18/21 (86)	21/21 (100)	14/15 (93)
Ao0 dilation on the total of available CTAs	0/18 (0)	3/21 (14)	0/21 (0)	1/15 (7)
Mean Ao0 variation – mm	0.14 (−2.9–2)	0.65 (−1.8–4.1)	0.08 (–4 - 2.3)	−0.58 (–5 - 1.9)

Data are shown as *n*/*N* (%) and mean (range). Ao0 = aortic neck diameter at the level of the lowest renal artery; CTA = computed tomography angiography.

### Secondary endpoints

**Figure 2:**
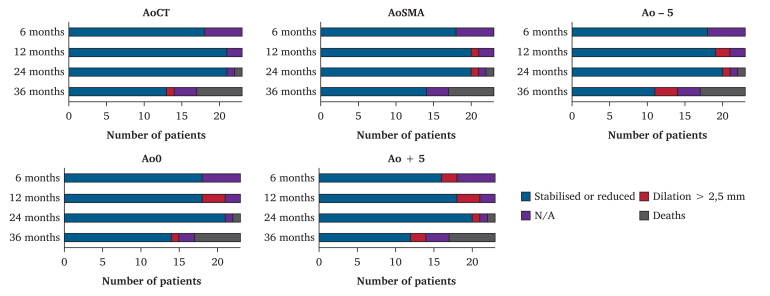
Aortic neck diameter variations after EndoSuture aneurysm repair at different levels of measurements during follow up. AoCT = central point of the ostium of the coeliac trunk; AoSMA = central point of the ostium of the superior mesenteric artery; Ao-5 = 5 mm above the inferior border of the ostium of the lower renal artery; Ao0 = at the inferior border of the lower renal artery; Ao+5 = 5 mm below the inferior border of the ostium of the lower renal artery; N/A = not available.

No major change in the diameters at the AoCT, AoSMA, Ao-5, and Ao+5 levels was observed during the three year follow up (Fig. 2). There was no rupture, persistent or new EL1a, type 3 endoleak, or limb occlusion during follow up. Three type 2 endoleaks (13%) were observed; two of these were present on post-operative CTA and resolved spontaneously during follow up. For one patient, it was persistent after two years, leading to an open sacotomy 987 days after ESAR; this patient died 248 days after this intervention due to spontaneous type A aortic dissection. Another patient was treated by total stent graft relining for a type 5 endoleak.

**Figure 3:**
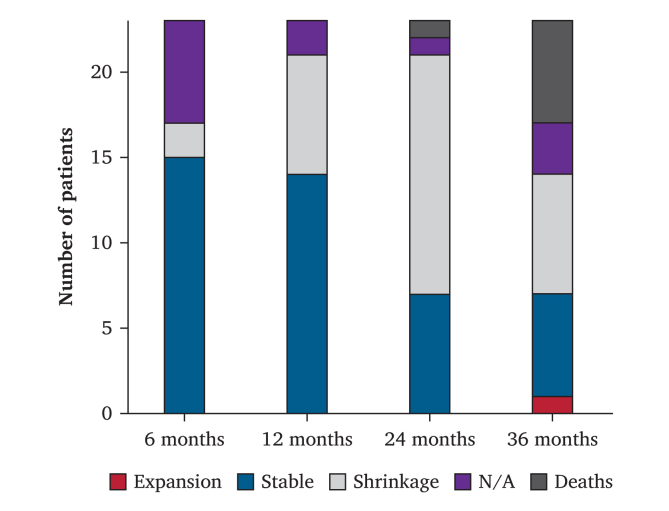
Aneurysm sac evolution during follow up after EndoSuture aneurysm repair. N/A = not available.

The AAA sac dynamics over three years are shown in Fig. 3. Among the 14 patients who completed their three year imaging follow up, the mean (range) variation in AAA sacs was −3.9 (−21.1–34.8) mm. Of those, seven (50%) had a >5 mm sac shrinkage, six (43%) had stable sacs, and one (7%) had a sac expansion of 34.8 mm leading to the open sacotomy mentioned above.

The 30 day and one year mortality rates were 0%. At three years, six patients (26%) had died: one from lung cancer, one from COVID-19 infection, one from myocardial infarction, one from cardiorespiratory arrest, one from metabolic complications due to malnutrition, and one from an unknown cause. In the latter case, no sac expansion or endoleak that could suggest an aortic related complication leading to death was observed on follow up imaging.

## Discussion

This retrospective, single centre cohort analysis of 23 patients confirmed the feasibility of ESAR in the primary treatment of AAA, with high technical and procedural success rates. In this highly selected population of patients with hostile aortic neck, and/or for whom elective open repair was contraindicated, and/or FEVAR could not be proposed, no aortic neck dilation and a high proportion of sac shrinkage was observed during the three year follow up. Aortic diameters remained stable at the suprarenal and infrarenal levels, without re-interventions for EL1a.

These findings are in line with data from the literature (Table 3), such as those from systematic reviews and meta-analyses,^6^^,^^7^ showing good short to midterm results, particularly in high technical success and freedom from EL1a following the primary deployment of EndoAnchors in the mid-^10^ and long term.^11^^,^^12^ Moreover, data from the literature have also previously underlined the positive effect of ESAR on sac shrinkage. The propensity score analysis by Muhs *et al.*^13^ found that ESAR leads to a higher rate of sac regression during the first two years compared with EVAR (81% *vs*. 49%). Similarly, Valdivia *et al.*^14^ reported higher rates of sac shrinkage (65% *vs*. 38%) and a higher proportion of patients achieving sac regression (59.4% *vs*. 32.3%) up to five years after ESAR. As proposed for EVAR, a one year assessment of AAA sac dynamics may predict late outcomes after ESAR, as sac regression was associated with lower mortality, re-intervention, and rupture rates within 8 years, whereas sac expansion was associated with higher rates of these outcomes.^15^ In the present cohort, AAA sac evolution after ESAR was satisfactory, as no sac expansion was observed during the first two years of follow up, and one expansion occurred at 36 months. In the latter case, despite the huge increase in sac diameter, no EL1a or migration occurred.

**Table 3:** Overview of published EndoSuture aneurysm repair mid- and long term results.

Author	Study type	Publication year	Number of patients	Mean age – years	Female – %	FU period/	Median FU – mo	Primary *vs*. secondary interventions – *n* (%)	Overall technical success – %	Freedom from all cause mortality – %	Freedom from aneurysm related mortality – %	Freedom from EL1a – %	Freedom from any endovascular or surgical procedure – %	Freedom from rupture – %	Aneurysm sac behaviour		
															Shrinkage – %	Stability – %	Expansion – %
Karaolanis *et al.*^7^	Meta-analysis	2020	968	N/A	41.8	N/A	10	742 *vs*. 127 (76.6 *vs*. 13.1%)	97.1	93.4	N/A	93.8	97.68	100	68.2	29.9	1.93
Qamhawi *et al.*^6^	Systematic review	2020	455	73.8∗	14.3	N/A	15.4	455 *vs*. 107 (76.5 *vs*. 23.5%)	98.4∗	N/A	N/A	96.5∗	29.89	N/A	55.3∗	43.5∗	1.4∗
Reyes-Valdivia *et al.*^11^	Registry	2022	221	75 ± 8.3	16.7	06/2010–12/2019	27 (range: 12–48)	175 *vs*. 46 (79 *vs*. 21%)	96.8	89	98	94	87	N/A	N/A	51	41
Arko III *et al.*^12^	Registry	2023	70	71.3 ± 8.1	27.1	2012-2023/5 years	Window day range: 1 645–2 009	N/A	N/A	68.5% ± 6.2	90.1 ± 4.5	N/A; 9 EL1a (3 spontaneously resolving; 3 endovascular reinterventions; 3 inoperable)	76.9 ± 7.2	95.6 ± 3.2	68.2	13.6	18.2
Abdel-Hadi *et al*.^10^	Single center retrospective study	2023	33∗ (4 TEVAR; 29 EVAR)	79 (median)	14	02/2017–03/2021	38 (2–71)	33 *vs*. 17 (66 *vs*. 33%)	98	95 at 1 year; 85 at 2 and 3 years	100	96 at 1 year; 93 at 2 and 3 years	97 at 1 year; 94 at 2 and 3 years FU∗	100	N/A	N/A	N/A
Ammoll*o et al*.	Single center retrospective study	2025	23	74.5 (median)	4.3	09/2017–05/2024	36.5 ± 16.31	23 *vs*. 0	100	74	100	100	91.30	100	50	42.8	7.2

TEVAR = thoracic endovascular aortic repair; FEVAR = fenestrated endovascular aortic repair; FU = follow up; EL1a = type 1A endoleaks; N/A = not available.

∗ Primary deployments. Aneurysm sac shrinkage was defined as a decrease of >5 mm, sac stability as a variation in a ±5 mm range, and sac expansion as an increase of >5 mm.

According to real world instructions for use, up to 60% of all EVAR may be considered complex, mainly due to neck lengths less than 10–15 mm.^16^ However, increasing operator experience and technical progress have broadened the applicability of endovascular strategies, such as fenestrated or branched stent grafts, EVAR outside instructions for use, physician modified stent grafts, as well as EVAR with adjuncts, such as chimneys and EndoAnchors.^17^^,^^18^ The standard of care for thoraco-abdominal, para-, and juxtarenal aortic aneurysms in this centre consists of FEVAR using the Anaconda fenestrated device (Terumo Aortic, Glasgow, Scotland), a strategy that has shown good midterm results.^19^ However, the anatomic (mainly iliac access and abdominal target vessel issues) and technical constraints (production delay for custom made stent grafts) related to FEVAR limit its applicability. Moreover, because of its higher short term morbidity and mortality rates, elective open repair is only proposed for young, fit patients with severe neck angulation, narrow iliac arteries, and hostile target vessels. Chimney stent grafts are exclusively performed in extremely urgent situations, such as ruptured juxtarenal aortic aneurysms or as a bail out for accidental renal artery coverage. Therefore, in patients with low life expectancy and contraindications to elective open repair and FEVAR, EVAR outside instructions for use with EndoAnchor fixation may be considered.

Studies have reported several advantages of EndoAnchor use, which seem to increase graft apposition to the aortic wall and may enable positive remodelling of the infrarenal neck with dramatically reduced migration rates compared with EVAR (0.26% *vs*. 0.7–6.3%).^20^, ^21^, ^22^, ^23^, ^24^ Such stability might be beneficial in the long term, as diminishing stent graft apposition by < 10 mm has recently been shown to be an important indicator of late EL1a after EVAR.^25^ However, as highlighted by the analysis of Gallitto *et al.* in a cohort of 129 patients with an infrarenal neck <10 mm, the overall feasibility of ESAR is poor (27%) compared with chimney EVAR or FEVAR (37% and 94%, respectively).^26^ The aortic wall penetration of EndoAnchors has been associated with several factors. According to Goudeketting *et al.*,^27^ the use of a Medtronic Endurant II/IIs stent graft was associated with better EndoAnchor penetration, whereas an aortic diameter 10 mm below the lowest renal artery and increased thickness of mural neck calcium were risk factors for poor penetration. Moreover, a study derived from the ANCHOR registry in patients undergoing ESAR for a revision of previous stent graft failure for EL1a indicated that 48% of EndoAnchors lacked adequate aortic wall penetration, suggesting that ESAR should probably be proposed only for primary deployments, as performed in the current centre.^13^ Finally, operator experience also appears to have a major impact on EndoAnchor penetration. Several studies have provided some interesting insights into the physical damage that EndoAnchors produce on the graft fabric. The main mechanism is the alteration of the textile structure either directly linked to several penetrations of the fabric by the same EndoAnchor, or tears of the textile fibres when the EndoAnchors pass through them, or through the interactions between EndoAnchors and graft stents.^14^ All these damages may also be amplified by improper use of the devices. In a recent *in vitro* study, the fabric damage by EndoAnchors seemed to be more extensive on expanded polytetrafluoroethylene (such as Endologix AFX2 or Gore Excluder) than on polyethylene terephthalate (Medtronic Endurant II/IIs or Cook Zenith), suggesting that such devices should be used according to instructions for use.^15^ The absence of EL1a and type 3 endoleak in the present cohort, however, suggests that these damages do not have major clinical or anatomic consequences. Moreover, all ESAR procedures were performed by the same experienced surgeon, exclusively using the Medtronic Endurant II/IIs graft, thereby probably minimising the complications linked to reduced aortic wall penetration and fabric damage. Importantly, ESAR remains a complex procedure, even in the hands of highly experienced endovascular surgeons, and should be considered as such.

This study had several limitations. The limited number of patients only allowed a descriptive analysis of the retrospectively collected data. Some degree of selection bias must also be considered, as ESAR was performed in highly selected patients (older, sicker patients with no indication for elective open repair or FEVAR). Moreover, all but one of the patients were men, with a median age of 75 years, introducing possible age and sex biases. Finally, at the last 36 month follow up, the results were heavily impaired by loss to follow up and deaths, since ESAR was proposed to older, frailer patients with limited overall life expectancy.

### Conclusion

This single centre analysis of aortic neck evolution three years after ESAR suggests that EndoAnchors may help prevent aortic neck and suprarenal aorta dilatation in the midterm, without re-interventions for type EL1a. ESAR is a feasible procedure in patients with hostile aortic neck, and/or who are unfit for open surgery, and/or in whom anatomical or technical constraints prevent the use of FEVAR. Further studies are required to confirm the long term safety of ESAR.

## Funding

The authors received no financial support for the research, authorship, or publication of this article.

## Conflict of interests

The authors declare no potential conflicts of interest with respect to the research, authorship, or publication of this article.
